# CGRP protects bladder smooth muscle cells stimulated by high glucose through inhibiting p38 MAPK pathway in vitro

**DOI:** 10.1038/s41598-021-87140-y

**Published:** 2021-04-07

**Authors:** Jun Xue, Yadong Liu, Sichong Zhang, Liucheng Ding, Baixin Shen, Yunpeng Shao, Zhongqing Wei

**Affiliations:** 1 Department of Urology, The Second Affiliated Hospital of Nanjing Medical University, 121 Jiangjiayuan Road, Nanjing, 210011 Jiangsu China; 2 Department of Urology, The Third People’s Hospital of Yancheng, Yancheng, 224001 Jiangsu China

**Keywords:** Cell biology, Drug discovery, Diseases, Medical research

## Abstract

This study aimed to explore the effect of calcitonin gene-related peptide (CGRP) on bladder smooth muscle cells (BSMCs) under high glucose (HG) treatment in vitro. BSMCs from Sprague–Dawley rat bladders were cultured and passaged in vitro. The third-generation cells were cultured and divided into control group, HG group, HG + CGRP group, HG + CGRP + asiatic acid (AA, p-p38 activator) group, CGRP group, AA group, HG + CGRP + CGRP-8-37 (CGRP receptor antagonist) group and HG + LY2228820 (p38 MAPK inhibitor) group. The cell viability, apoptosis, malondialdehyde (MDA) and superoxide dismutase (SOD) levels of BSMCs were observed by the relevant detection kits. The expressions of α-SM-actin, p38 and p-p38 were detected by qRT-PCR or Western blot analysis. Compared with the control group, the cell viability, SOD and α-SM-actin levels of BSMCs were decreased and apoptotic cells, MDA and p-p38 levels were increased after HG treatment, while these changes could be partly reversed when BSMCs were treated with HG and CGRP or LY2228820 together. Moreover, AA or CGRP-8-37 could suppress the effect of CGRP on BSMCs under HG condition. Our data indicate that CGRP protects BSMCs from oxidative stress induced by HG in vitro, and inhibit the α-SM-actin expression decrease through inhibiting the intracellular p38 MAPK signaling pathway.

## Introduction

Diabetic cystopathy (DCP) is a common urinary complication of diabetes. Even in patients with stable glycemic control, the incidence of DCP is still as high as 25%^1^. Patients with DCP mainly present with symptoms such as low bladder detrusor contractility and decreased urination sensation, which eventually causes chronic urinary retention and full urinary incontinence^2, 3^.

The exact pathogenesis of DCP is not fully understood. The etiology research is currently focused on three parties: myogenic factors, neurogenic factors and oxidative stress. Oxidative stress responses induced by hyperglycemia are increasingly becoming the focus of research^4–6^. The consequence of hyperglycemic stimulation is the increase of reactive oxygen species (ROS) and reactive nitrogen species (RNS), thus starting the oxidative stress. These reactive molecules can directly oxidize and damage DNA, proteins and lipids, which can also activate multiple stress-sensitive signaling pathways in cells as signaling molecules^7–9^. At present, it is believed that oxidative stress causes peripheral autonomic neuropathy and bladder smooth muscle damage, resulting in progressively worsening bladder function^10–12^. Elrashidy et al. has reported that long-term diabetes caused oxidative stress that could promote the bladder muscle fibrosis and apoptosis^11^. Calcitonin gene-related peptide (CGRP) is the main constituent neuropeptide of sensory nerves. Our previous studies showed CGRP was down-regulated in DCP, which contributes to the bladder dysfunction^13, 14^. Other research has also indicated that the content of CGRP in the bladder wall of diabetic rats significantly decreased, suggesting that CGRP plays a key role in the development of DCP^15^. It is reported that CGRP can decrease the oxidative stress level in rats with severe acute pancreatitis through inhibiting the p38 MAPK pathway activity^16^. Our previous study also showed that CGRP could decrease the oxidative stress level in dorsal root ganglion neurons under high glucose (HG) treatment in vitro through PI3K/AKT pathway^14^.

We speculated that CGRP protected bladder smooth muscle cells (BSMCs) from oxidative stress induced by HG in vitro. In the current study, the cell model with diabetes was induced with HG, then the cell viability, apoptosis and α-SM-actin (contractile marker in smooth muscle cells) expression of BSMCs under HG with or without CGRP in vitro were observed, and then asiatic acid (AA, p-p38 activator), CGRP-8-37 (CGRP receptor antagonist) and LY2228820 (p38 MAPK inhibitor) were applied to explore the possible relevant mechanism.

## Materials and methods

### Animal

The experiment was approved by Ethical Committee on Animal Experiment Committee of Nanjing Medical University (KY-20190643). Sprague–Dawley rats (n = 12, male 6, female 6; 200–220 g, 213.58 ± 5.84 g) were purchased from Nanjing Medical University Animal Experiment Center. All experiments and methods were performed in accordance with the Animal Research: Reporting of In Vivo Experiments (ARRIVE) guidelines^17^. We declared that all methods were carried out in accordance with the relevant guidelines and regulations.

### Cell culture

The rat bladder smooth muscle cells (BSMCs) were isolated and cultured according to previous studies^18–21^. Briefly, the rat was anesthetized and the bladder was removed quickly. The bladder was rinsed with 0.01 M sterile phosphate buffered saline (PBS) and minced into 1 mm^3^ sections, then was incubated with 0.3 mg/ml soybean trypsin inhibitor, 1 mg/ml collagenase I, 0.2 mg/ml elastase III and 2 mg/ml crystallized bovine serum albumin for 1.5 h at 37 ℃. The cells were cultured in Dulbecco's modified Eagle's medium (DMEM) containing 5 mM D-glucose (Gibco/BRL), 100 U/ml penicillin, 15% fetal bovine serum (FBS) and 100 mg/ml streptomycin. The culture medium was replaced with fresh medium twice a week. The cells were passaged when they had reached 80–90% confluence, and the third- generation cells were used in the following experiments.

### Cell treatments

The cell model with diabetes was induced by HG as previously described by Qiu^22, 23^. Briefly, BSMCs were grown in DMEM supplemented with 5 mM D-glucose (normal glucose as a control), to mimic diabetes in vitro, the cells were maintained in DMEM with 25 mM D-glucose (Gibco/BRL) for 48 h as a HG treatment. BSMCs were divided into 8 groups: control group, HG group, HG + CGRP group, HG + CGRP + AA group, CGRP group, AA group, HG + CGRP + CGRP-8-37 group and HG + LY2228820 group. The relevant use concentrations were as follows: CGRP (20 pg/ml, Abcam, UK), AA (5 μM, Abcam, UK), CGRP-8-37 (2 μM, Abcam, UK) and LY2228820 (1 μM, Abcam, UK). The cells were incubated in the incubator with 5% CO_2_ at 37 °C for 48 h.

### Methyl-thiazole-tetrazolium (MTT) assay

The cell viability of the cultured BSMCs was measured by MTT kit (Amresco, USA) according to the manufacturer’s instructions.

### Terminal deoxynucleotidyl transferase-mediated dUTP nick end labeling (TUNEL)

The apoptosis of BSMCs was measured using TUNEL kit for rats (Beyotime Biotechnology, China) according to the manufacturer’s instructions.

### Malondialdehyde (MDA) and superoxide dismutase (SOD) levels

The MDA and SOD levels in BSMCs were detected by MDA kit (Beyotime Biotechnology, China) and SOD kit (Beyotime Biotechnology, China) respectively according to the manufacturer’s instructions.

### Immunofluorescence

Cell culture medium was replaced with 5% goat serum to block non-specific reaction at room temperature for 30 min and then the cells were incubated with 1:500 diluted rabbit anti-α-SM-actin primary antibody (Abcam, UK) for 6 h at room temperature. Then the cells were washed three times with 0.01 M PBS and incubated with 1:500 diluted Alexa Fluor 488-conjugated goat anti-rabbit second antibody (Abcam, UK) in the dark for 3 h at room temperature. Hoechst33342 was used to stain the cell nuclei in the dark for 0.5 h at room temperature. The α-SM-actin immunopositive cells were observed and photographed with a fluorescent microscope (Leica DMIRB, Germany).

### Quantitative real-time PCR (qRT-PCR) analysis

The qRT-PCR was performed according to previous study^24^. The Trizol reagent (Invitrogen) was used to extract the total RNA of BSMCs in the four groups. The cDNA was generated with RevertAidTM First Strand cDNA Synthesized Kit (Fermentas, Canada). The PCR analyses were conducted using FastStart Universal SYBR Green Master Mix (Roche, Switzerland) on Corbett RG-6000 PCR system (QIAGEN, German). The sense and antisense primers were synthesized as follows: p38 forward 5′-AGGAGAGGCCCACGTTCTAC-3′, reverse 5′-TCAGGCTCTTCCATTCGTCT-3′; α-SM-actin forward 5′-GGAAGACAGCACAGCTCTGG-3′, reverse 5′-CATAGAGGGACAGCACAGCC-3′; GAPDH forward 5′-GCAAGTTCAACGGCACAG-3′, reverse 5′-GCCAGTAGACTCCACGACAT-3′. GAPDH was used as an internal control. The results were calculated with comparative C_t_ method.

### Western blot analysis

Western blot analysis was applied to determine the levels of protein expression in cells according to previous studies^25–28^. Briefly, the proteins in the four groups were extracted by RIPA buffer (Beyotime, China) and transfected onto the PVDF membranes, then the PVDF membranes were incubated with 1:500 diluted mouse anti-p38 primary antibody (Abcam, UK), 1:500 diluted mouse anti-p-p38 primary antibody (Santa Cruz, USA), 1:500 diluted mouse anti-α-SM-actin primary antibody (Abcam, UK) and 1:1000 diluted rabbit anti-β-actin primary antibody (Sigma-Aldrich Co., USA) overnight at 4 °C. The following day, the membranes were probed with the 1:5000 IRDye diluted 700-conjugated affinity-purified goat anti-mouse second antibody (Rockland Immunochemicals, USA) or 1:5000 diluted IRDye 800-conjugated affinity-purified goat anti-rabbit second antibody (1:5000, Rockland Immunochemicals, USA) for 60 min at room temperature. Then protein bands were visualized using Odyssey laser scanning system (LI-COR Inc., USA) and the intensities were quantified by Odyssey 3.0 image analysis system software.

### Statistical analysis

Data were expressed as mean ± standard deviation (M ± SD). The statistics package for social science 21.0 (SPSS 21.0) was used to do the statistical analysis. The differences among the groups were analyzed by one-way analysis of variance (ANOVA). Statistical charts are made with GraghPad Prism 7.0 software. The difference at *p* < 0.05 was considered statistically significant.

### Ethics approval

The experiment was approved by Ethical Committee on Animal Experiment Committee of Nanjing Medical University.

## Results

### The cell viability and apoptosis of BSMCs

**Figure 1:**
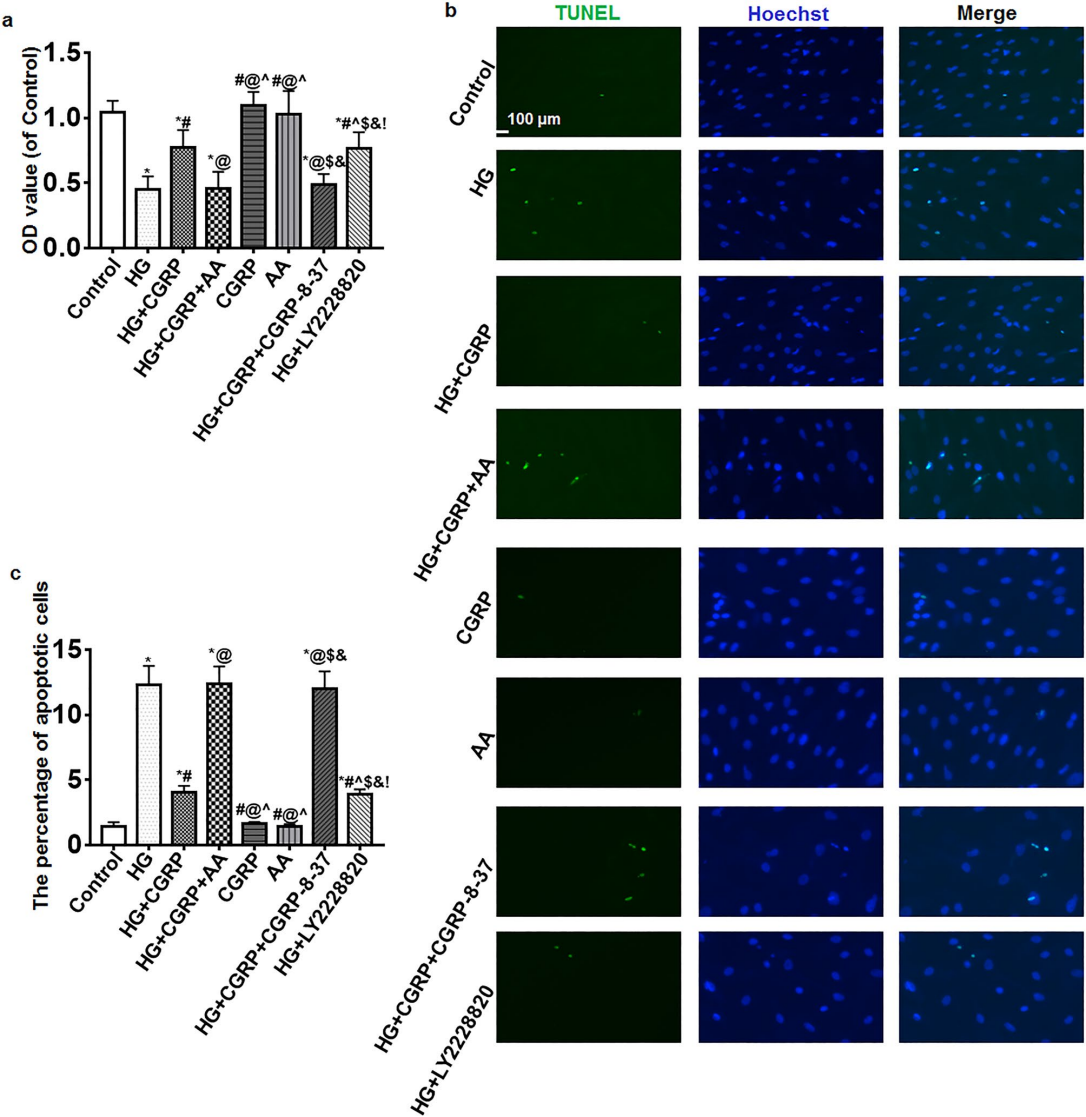
(**a**) The viability of BSMCs was detected by MTT. Compared with the control, CGRP and AA groups, the OD values of the other groups decreased but the OD values of the HG + CGRP and HG + LY2228820 groups were higher than that in the HG, HG + CGRP + AA and HG + CGRP + CGRP-8-37 groups. (**b**) TUNEL assay results showed that there were almost no apoptotic cells in the control, CGRP and AA groups. More apoptotic cells were observed in the HGgroup, after CGRP or LY2228820 treatment, the number of apoptotic cells was decreased. AA or CGRP-8-37 reversed the effect of CGRP on the cells. (**c**) Statistical histogram of the percentage of apoptotic cells in each group. *VS. control group, *p* < 0.05; ^#^ VS. HG group, *p* < 0.05; ^@^ VS. HG + CGRP group, *p* < 0.05; ^ VS. HG + CGRP + AA group, *p* < 0.05; $ VS. CGRP group, *p* < 0.05; & VS. AA group, *p* < 0.05; ! VS. HG + CGRP + CGRP-8-37 group, *p* < 0.05. Bar = 100 μm. n = 6. The bar charts were created by GraghPad Prism 7.0 software (Version 7.00, URL: www.graphpad.com).

The viability of BSMCs was detected by MTT. Compared with the control, CGRP and AA groups, the OD values of the other groups decreased but the OD values of the HG + CGRP and HG + LY2228820 groups were higher than that in the HG, HG + CGRP + AA and HG + CGRP + CGRP-8-37 groups (*p* < 0.05) (Fig. 1a).

The apoptosis of BSMCs was detected by TUNEL. Results showed that there were almost no apoptotic cells in the control, CGRP and AA groups. More apoptotic cells were observed in the HG group, after CGRP or LY2228820 treatment, the number of apoptotic cells was decreased. AA or CGRP-8-37 reversed the effect of CGRP on the cells. The difference of percentage of apoptotic cells was statistically significant (*p* < 0.05) (Fig. 1b, c).

### Oxidative stress level of BSMCs

**Figure 2:**
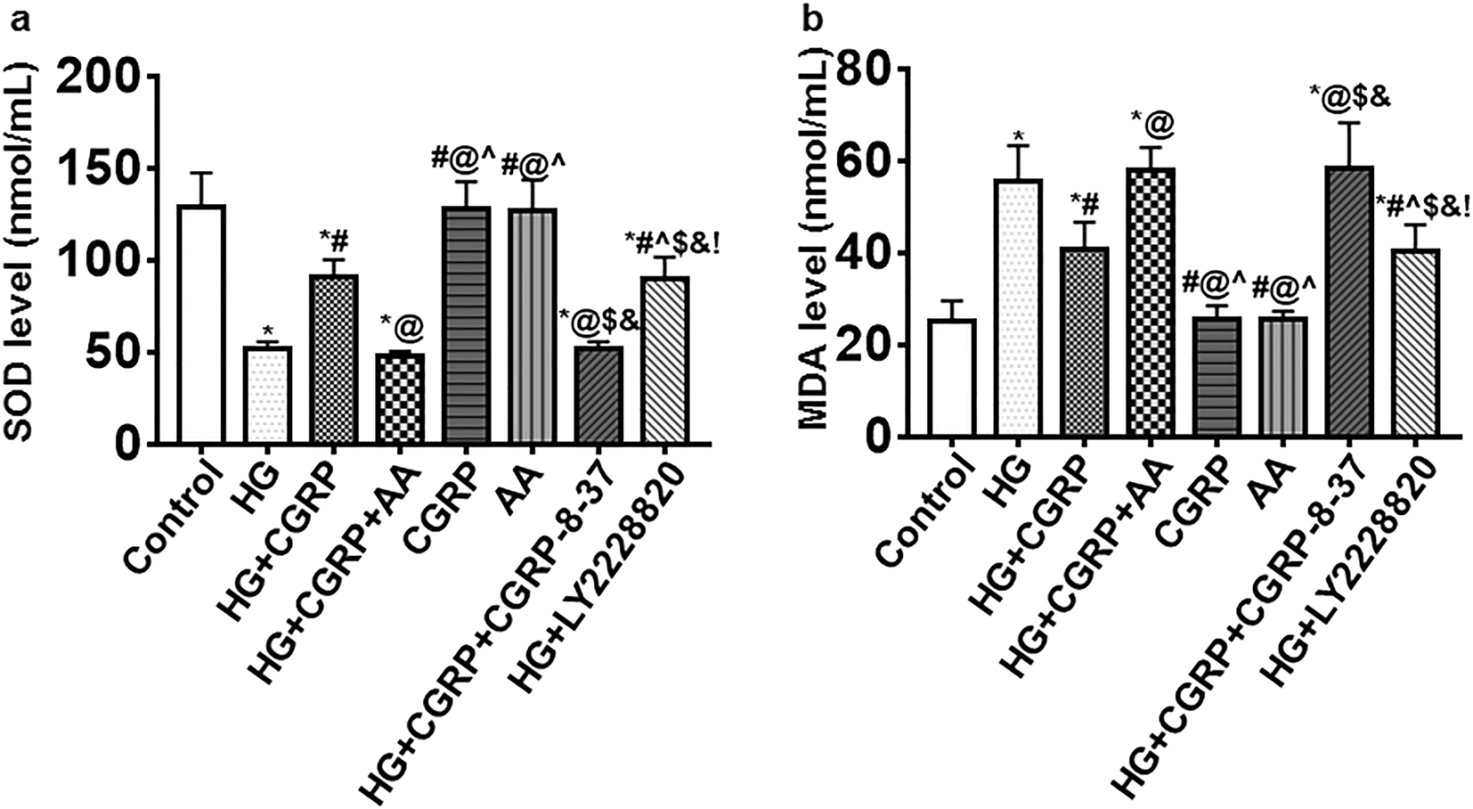
The oxidative stress level of BSMCs was measured by SOD and MDA kits. (**a**) Compared with the control, CGRP and AA groups, the SOD levels in the other groups decreased, but the SOD levels in the HG + CGRP and HG + LY2228820 groups were higher than that in the HG group, HG + CGRP + AA and HG + CGRP + CGRP-8-37 groups. (**b**) Compared with the control, CGRP and AA groups, the MDA level in the other groups increased but the MDA levels in the HG + CGRP and HG + LY2228820group were lower than that in the HG group, HG + CGRP + AA and HG + CGRP + CGRP-8-37 groups. * VS. control group, *p* < 0.05; ^#^ VS. HG group, *p* < 0.05; ^@^ VS. HG + CGRP group, *p* < 0.05; ^ VS. HG + CGRP + AA group, *p* < 0.05; $ VS. CGRP group, *p* < 0.05; & VS. AA group, *p* < 0.05; ! VS. HG + CGRP + CGRP-8-37 group, *p* < 0.05. n = 6. The bar charts were created by GraghPad Prism 7.0 software (Version 7.00, URL: www.graphpad.com).

Compared with the control, CGRP and AA groups, the SOD levels in the other groups decreased, but the SOD levels in the HG + CGRP and HG + LY2228820 groups were higher than that in the HG group, HG + CGRP + AA and HG + CGRP + CGRP-8-37 groups (*p* < 0.05) (Fig. 2a).

Compared with the control, CGRP and AA groups, the MDA level in the other groups increased but the MDA levels in the HG + CGRP and HG + LY2228820group were lower than that in the HG group, HG + CGRP + AA and HG + CGRP + CGRP-8-37 groups (*p* < 0.05) (Fig. 2b).

### The expression of α-SM-actin in BSMCs

**Figure 3:**
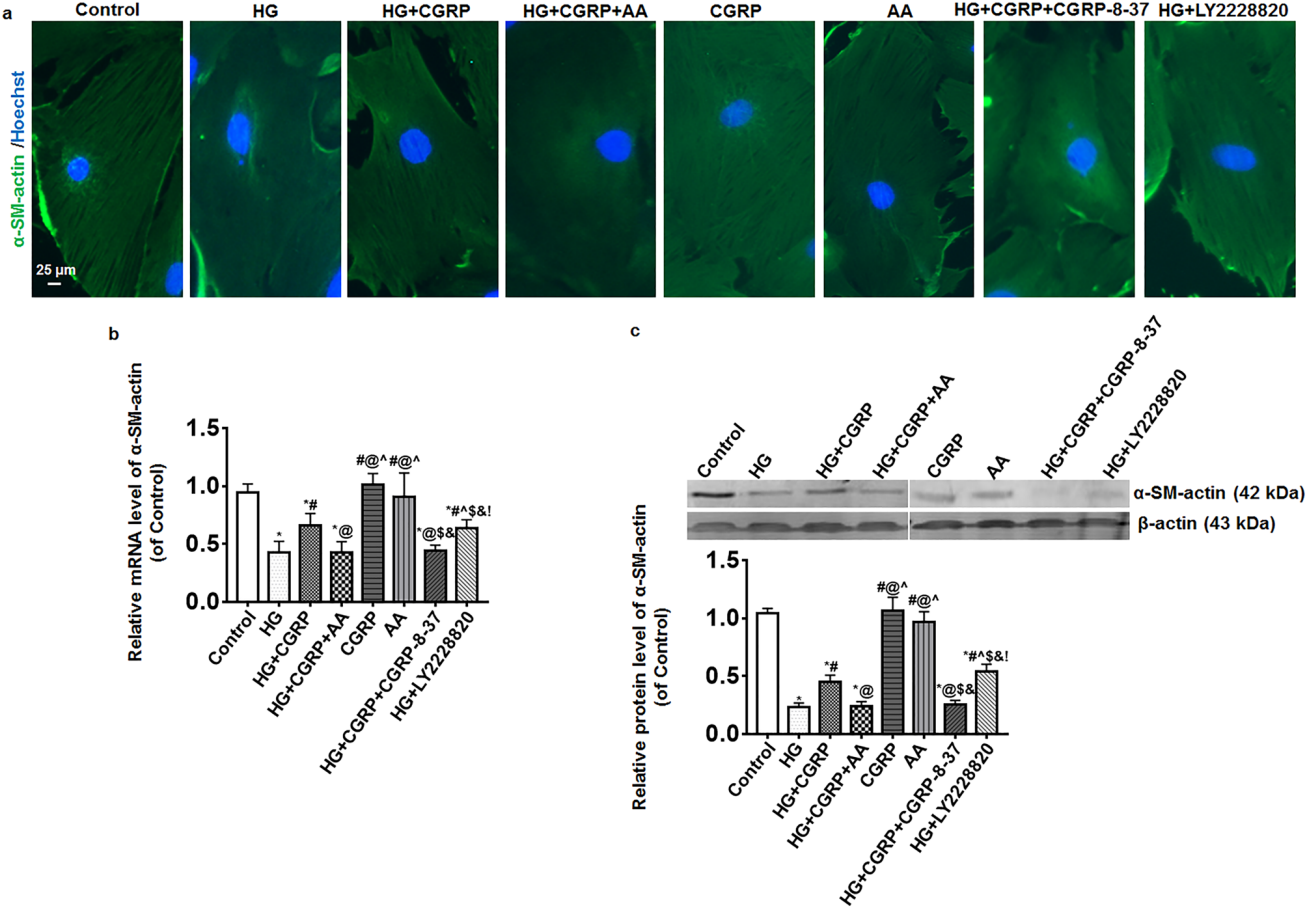
The expression of α-SM-actin in BSMCs was measured by immunofluorescence, qRT-PCR and Western blot. (**a**) The result of immunofluorescence showed the visible α-SM-actin fibers in BSMCs were found in the control, CGRP, AA, HG + CGRP and HG + LY2228820 groups while they were unclear in the HG, HG + CGRP + AA and HG + CGRP + CGRP-8-37 groups. (**b**,**c**) Compared with the control, CGRP and AA groups, the mRNA and protein expressions of α-SM-actin in the HG group, the HG + CGRP + AA and HG + CGRP + CGRP-8-37 groups were significantly decreased. Though the expression of α-SM-actin mRNA and protein in the HG + CGRP and HG + LY2228820 groups also deceased, it was higher than that in the HG, HG + CGRP + AA and HG + CGRP + CGRP-8-37 groups. * VS. control group, *p* < 0.05; ^#^ VS. HG group, *p* < 0.05; ^@^ VS. HG + CGRP group, *p* < 0.05; ^ VS. HG + CGRP + AA group, *p* < 0.05; $ VS. CGRP group, *p* < 0.05; & VS. AA group, *p* < 0.05; ! VS. HG + CGRP + CGRP-8-37 group, *p* < 0.05. Bar = 25 μm. n = 6. The bar charts were created by GraghPad Prism 7.0 software (Version 7.00, URL: www.graphpad.com). The Western blot bands were created by Odyssey 3.0 image analysis system software (Version 1.2.15, URL: www.licor.com/bio/supportsupport).

The expression of α-SM-actin in BSMCs was measured by immunofluorescence, qRT-PCR and Western blot. The result of immunofluorescence showed the visible α-SM-actin fibers of BSMCs were found in the control, CGRP, AA, HG + CGRP and HG + LY2228820 groups while they were unclear in the HG, HG + CGRP + AA and HG + CGRP + CGRP-8-37 groups (Fig. 3a). Compared with the control, CGRP and AA groups, the mRNA expressions of α-SM-actin in the HG group, the HG + CGRP + AA and HG + CGRP + CGRP-8-37 groups were significantly decreased. Though the expression of α-SM-actin mRNA in the HG + CGRP and HG + LY2228820 groups also deceased, it was higher than that in the HG, HG + CGRP + AA and HG + CGRP + CGRP-8-37 groups (*p* < 0.05) (Fig. 3b). The change of the protein expression of α-SM-actin among the groups was similar to that of mRNA (*p* < 0.05) (Fig. 3c).

### The expression of p38 MAPK in BSMCs

**Figure 4:**
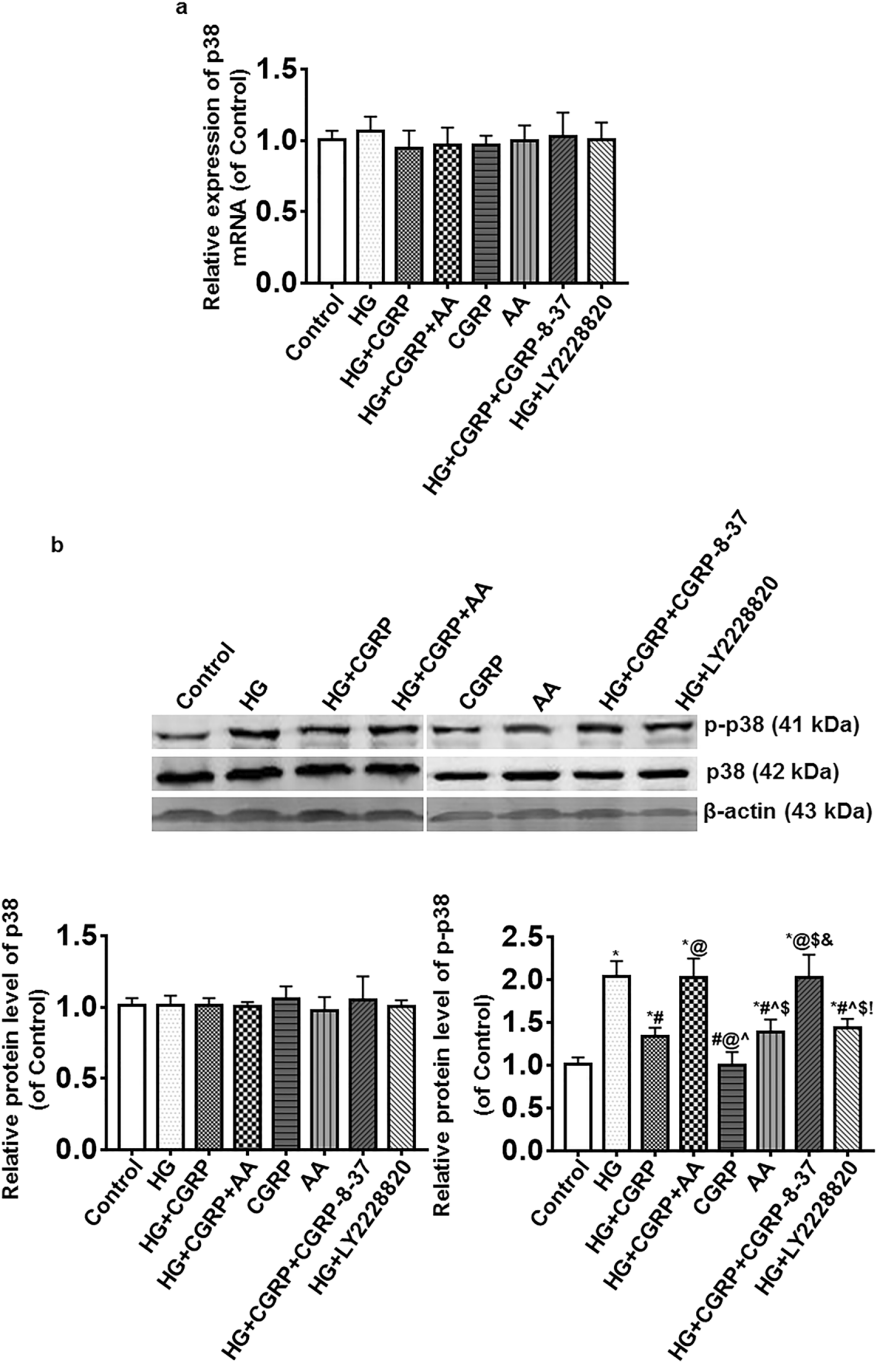
The expressions of p38 MAPK in BSMCs were detected by qRT-PCR and Western blot. (**a**) The expression of p38 mRNA in the groups was no significant change. (**b**) The expression of p38 protein in the groups was no significant change. Compared with the control and CGRP groups, the expression of p-p38 protein in AA group increased. The expression of p-p38 protein in the HG group was notably increased, the degree of the change was reduced when BSMCs were treated with CGRP or LY2228820 at the same time, and AA or CGRP-8-37 could suppress the effect of CGRP on the expression of p-p38 protein in BSMCs under HG. . * VS. control group, *p* < 0.05; ^#^ VS. HG group, *p* < 0.05; ^@^ VS. HG + CGRP group, *p* < 0.05; ^ VS. HG + CGRP + AA group, *p* < 0.05; $ VS. CGRP group, *p* < 0.05; & VS. AA group, *p* < 0.05; ! VS. HG + CGRP + CGRP-8-37 group, *p* < 0.05. n = 6. The bar charts were created by GraghPad Prism 7.0 software (Version 7.00, URL: www.graphpad.com). The Western blot bands were created by Odyssey 3.0 image analysis system software (Version 1.2.15, URL: www.licor.com/bio/supportsupport).

The expressions of p38 mRNA and protein in the groups were no significant changes (*p* > 0.05). Compared with the control and CGRP groups, the expression of p-p38 protein in the AA group increased. The expression of p-p38 protein in the HG group was notably increased, the degree of the change was reduced when BSMCs were treated with CGRP or LY2228820 at the same time, and AA or CGRP-8-37 could suppress the effect of CGRP on the expression of p-p38 protein in BSMCs under HG (Fig. 4).

## Discussion

Patients with DCP mainly present low bladder detrusor contractility, however, the effect of hyperglycemia on BSMCs remains largely elusive. To explore the possible related mechanism, the cell model with diabetes was induced with HG in this research according to previous studies^22, 23^. At the beginning, BSMCs were cultured and passaged in the medium containing 5 mM D-glucose, to mimic diabetes in vitro, and then BSMCs were maintained in the medium with 25 mM D-glucose for 48 h. Results showed that the viability of BSMCs significantly decreased and apoptotic cells became more after HG treatment, at the same time the SOD level decreased and MDA increased. SOD is an important antioxidant enzyme, and its level decreases, suggesting a decline in antioxidant capacity^29^. MDA is a lipid oxidative damage marker, and its increased level indicates a higher level of oxidative stress^30^. The above results indicate that the BSMC model with diabetes has been successfully established and HG caused the BSMC damage through oxidative stress.

The α-SM-actin is the main contractile marker of smooth muscle^31, 32^. The effect of hyperglycemia on the expression of α-SM-actin is still unclear. In this research, the expression of α-SM-actin in BSMC model with diabetes was measured by immunofluorescence, qRT-PCR and Western blot, the results displayed that the expression of α-SM-actin in BSMCs induced by HG significantly decreased, which may attribute to low bladder detrusor contractility in DCP.

It is well known that the MAPK signaling pathway plays an important role in the regulation of cell migration, proliferation and apoptosis, among them, ERK1/2 cascades are mostly activated for cell growth factor-stimulated signaling, whereas JNK and p38 MAPK process cell stress evoked by physical, chemical and biological stress stimuli^33, 34^. Our results showed that p-p38 protein level was dramatically increased in BSMCs of HG group and p38 MAPK inhibitor LY2228820 could reduce the change, which suggests that p38 MAPK signaling pathway is involved in BSMC damage through oxidative stress. Many studies have shown that diabetes can cause a decrease in CGRP, CGRP declines in the state of hyperglycemia, causing strong and long-lasting contraction of blood vessels, resulting in vascular endothelial damage, microcirculation disorders, tissue ischemia and hypoxia, which contribute to various diabetes complications, and many agents targeting the CGRP system are in clinical trials^35–38^. CGRP are the main transmitters affiliated with bladder sensory nerves—specifically, the content of CGRP in the bladder wall of diabetic rats, especially in the submucosal plexus, and the CGRP nerve distribution significantly decreased, suggesting that CGRP plays a key role in the development of DCP^15^. Some studies show CGRP protects cells or animals from oxidative stress through the MAPK signaling pathway^39–42^. In the current study, after BSMC model with diabetes induced by HG was treated with CGRP or LY2228820, the cell viability and α-SM-actin expression increased, and apoptosis, oxidative stress and p-p38 protein level decreased. These results indicate that CGRP protects BSMCs from oxidative stress through inhibiting p38 MAPK signaling pathway, and to further verify it, the BSMC model with diabetes was treated by CGRP and p-p38 activator or CGRP receptor antagonist together, and AA or CGRP-8-37 could suppress the protective effect of CGRP.

## Conclusions

CGRP protects BSMCs under HG condition in vitro from oxidative stress, and inhibit the α-SM-actin expression decrease through inhibiting the intracellular p38 MAPK signaling pathway.

## Supplementary Information

Supplementary Information

## Data Availability

The primary data are available in the “Supplementary Material”.

## Code availability

N/A.
