# Supplementary material for: CGRP protects bladder smooth muscle cells stimulated by high glucose through inhibiting p38 MAPK pathway in vitro
Source: Sci Rep. 2021 Apr 7;11:7643. doi: 10.1038/s41598-021-87140-y (PMC8027675; doi:10.1038/s41598-021-87140-y)
Supplement: Supplementary file 1 — Supplementary Information [file 41598_2021_87140_MOESM1_ESM.pptx]

## Slide 1
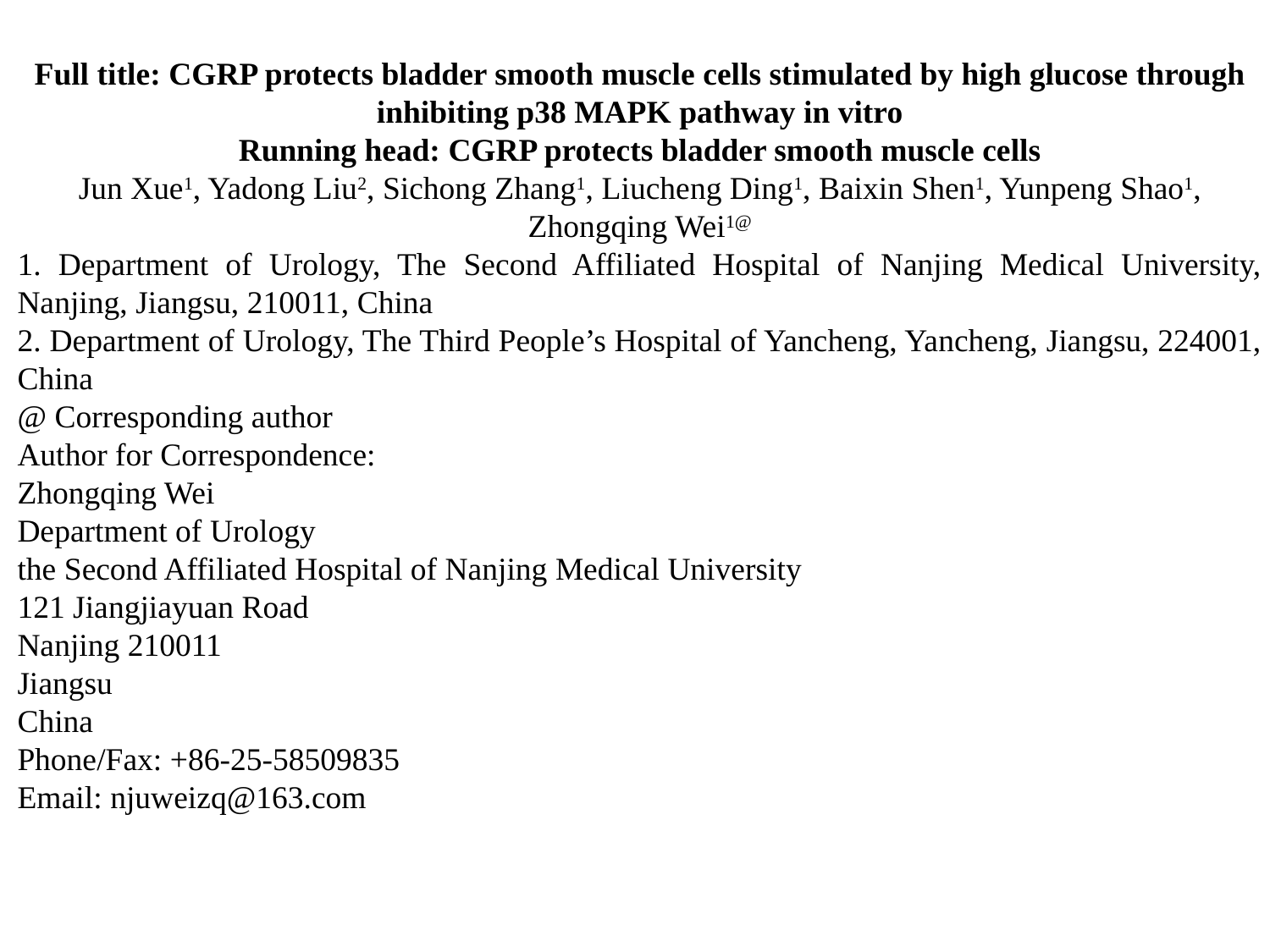

Full title: CGRP protects bladder smooth muscle cells stimulated by high glucose through inhibiting p38 MAPK pathway in vitro
Running head: CGRP protects bladder smooth muscle cells
Jun Xue1, Yadong Liu2, Sichong Zhang1, Liucheng Ding1, Baixin Shen1, Yunpeng Shao1, Zhongqing Wei1@
1. Department of Urology, The Second Affiliated Hospital of Nanjing Medical University, Nanjing, Jiangsu, 210011, China
2. Department of Urology, The Third People’s Hospital of Yancheng, Yancheng, Jiangsu, 224001, China
@ Corresponding author
Author for Correspondence:
Zhongqing Wei
Department of Urology
the Second Affiliated Hospital of Nanjing Medical University
121 Jiangjiayuan Road
Nanjing 210011
Jiangsu
China
Phone/Fax: +86-25-58509835
Email: njuweizq@163.com

## Slide 2
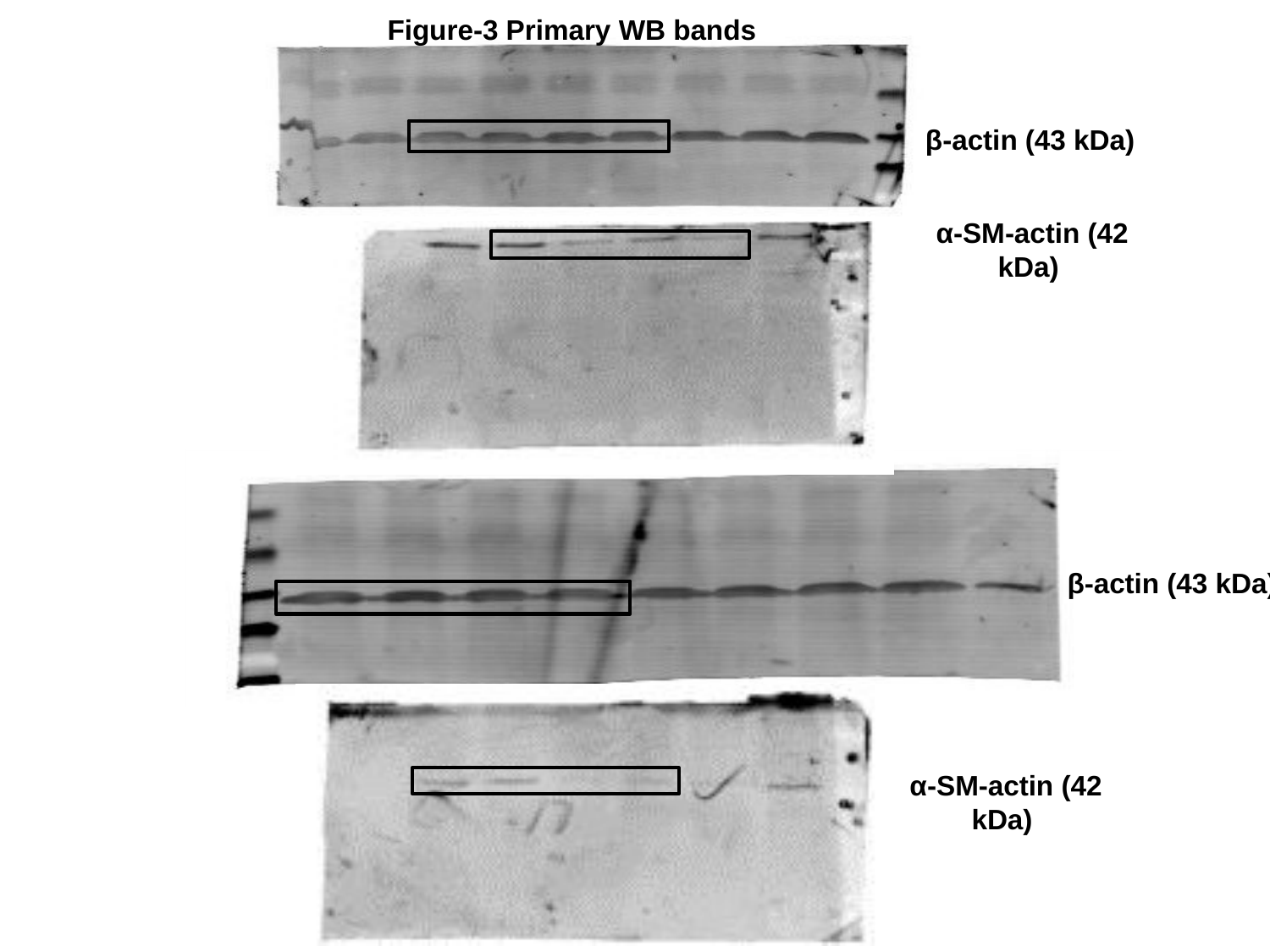

Figure-3 Primary WB bands
β-actin (43 kDa)
α-SM-actin (42 kDa)
β-actin (43 kDa)
α-SM-actin (42 kDa)

## Slide 3
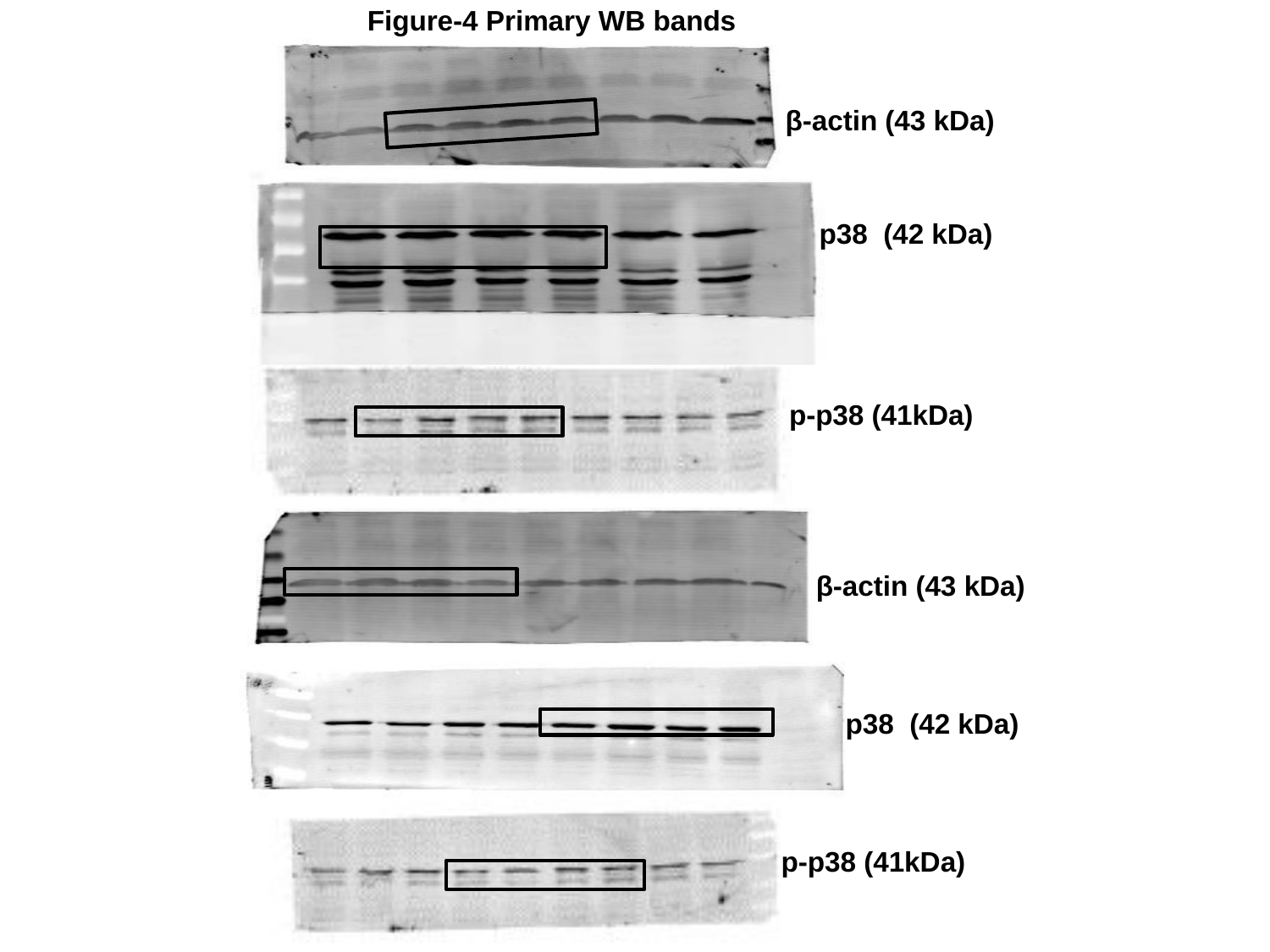

Figure-4 Primary WB bands
β-actin (43 kDa)
p38 (42 kDa)
p-p38 (41kDa)
β-actin (43 kDa)
p38 (42 kDa)
p-p38 (41kDa)

## Slide 4
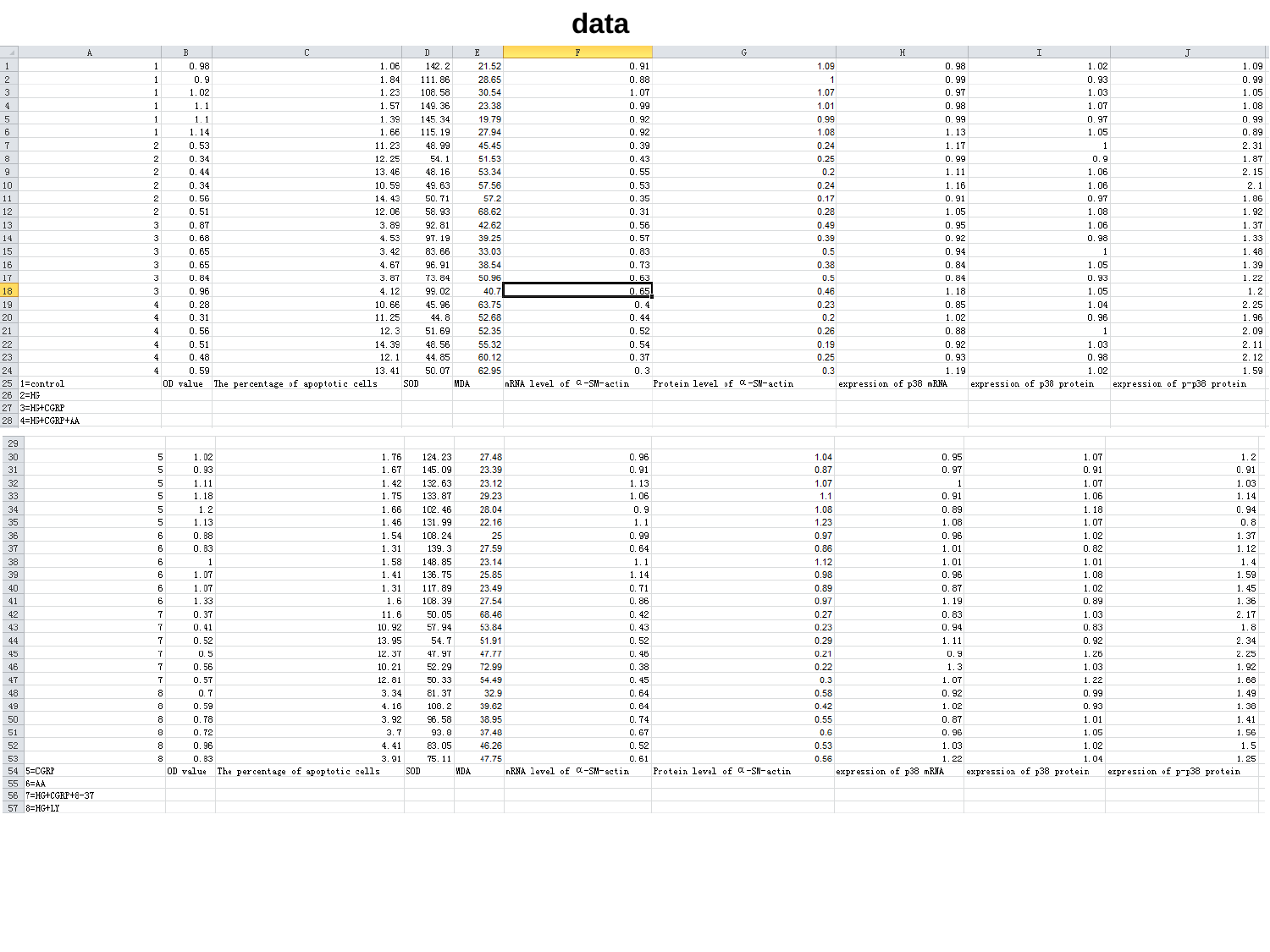

data
